# Local Structures of Two-Dimensional Zeolites—Mordenite and ZSM-5—Probed by Multinuclear NMR

**DOI:** 10.3390/molecules25204678

**Published:** 2020-10-14

**Authors:** Marina G. Shelyapina, Rosario I. Yocupicio-Gaxiola, Iuliia V. Zhelezniak, Mikhail V. Chislov, Joel Antúnez-García, Fabian N. Murrieta-Rico, Donald Homero Galván, Vitalii Petranovskii, Sergio Fuentes-Moyado

**Affiliations:** 1 Department of Nuclear Physics Research Methods, Saint-Petersburg State University, 7/9 Universitetskaya nab., St. Petersburg 199034, Russia; st022650@student.spbu.ru (I.V.Z.); mikhail.chislov@spbu.ru (M.V.C.); 2 Center for Scientific Research and Higher Education at Ensenada (CICESE), Ensenada, Baja California 22860, Mexico; ryocu@cnyn.unam.mx; 3 Center for Nanoscience and Nanotechnology, National Autonomous University of Mexico (CNyN, UNAM), Ensenada, Baja California 22860, Mexico; joel.antunez@gmail.com (J.A.-G.); fabian.murrieta@uabc.edu.mx (F.N.M.-R.); donald@cnyn.unam.mx (D.H.G.); vitalii@cnyn.unam.mx (V.P.); fuentes@cnyn.unam.mx (S.F.-M.)

**Keywords:** lamellar 2D zeolites, pillared zeolites, mordenite, ZSM-5, CTAB, NMR

## Abstract

Mesostructured pillared zeolite materials in the form of lamellar phases with a crystal structure of mordenite (MOR) and ZSM-5 (MFI) were grown using CTAB as an agent that creates mesopores, in a one-pot synthesis; then into the CTAB layers separating the 2D zeolite plates were introduced by diffusion the TEOS molecules which were further hydrolyzed, and finally the material was annealed to remove the organic phase, leaving the 2D zeolite plates separated by pillars of silicon dioxide. To monitor the successive structural changes and the state of the atoms of the zeolite framework and organic compounds at all the steps of the synthesis of pillared MOR and MFI zeolites, the nuclear magnetic resonance method (NMR) with magic angle spinning (MAS) was applied. The ^27^Al and ^29^Si MAS NMR spectra confirm the regularity of the zeolite frameworks of the as synthetized materials. Analysis of the ^1^H and ^13^C MAS NMR spectra and an experiment with variable contact time evidence a strong interaction between the charged “heads” –[N(CH_3_)_3_]^+^ of CTAB and the zeolite framework at the place of [AlO_4_]^−^ location. According to ^27^Al and ^29^Si MAS NMR the evacuation of organic cations leads to a partial but not critical collapse of the local zeolite structure.

## 1. Introduction

In recent years, much attention has been paid to the techniques of the “one-pot synthesis” for the direct production of zeolitic materials. Zeolites are undoubtedly important heterogeneous catalysts, and the number of industrial processes, in which they are used in that capacity, has been constantly increasing. The interest is mainly issued by the great opportunities these methodologies open up when developing functional zeolite based materials, such as hybrid organic-inorganic molecular sieves [1,2,3], hierarchical microporous-mesoporous zeolites [4,5,6,7], nanozeolites [8,9,10], and template-free molecular sieves [4,7,11].

Zeolites with hierarchical porous structure can also be synthesized directly, without using templates. The template-free methods are mainly based on the following strategies [7]: (i) the development of intercrystalline mesoporosity due to aggregation of nanocrystals; (ii) the emergence of intracrystalline mesoporosity, which is formed by amorphous gels that control crystallization; (iii) mesoporosity created between self-pillared two-dimensional zeolite nanosheets. The latter can be obtained by synthesizing layered zeolites in the presence of organic structure directing agents (OSDA) followed by calcination [4]. It is known that the use of various OSDA makes it possible to obtain target zeolites with specific physicochemical properties, and even novel or improved zeolitic frameworks [12]. In this sense, the physicochemical properties of zeolites are highly dependent on the synthesis procedure, including the choice of OSDA. The latter is of primary importance for the aluminum distribution, the acidic properties of the obtained material, the size and morphology of crystals, which are the key parameters for the catalysts [13,14,15,16].

One of the widely used OSDA for the synthesis of mesoporous silica and organic-inorganic layered materials is Cetyltrimethylammonium Bromide (CTAB). It is also widely applied in the synthesis of zeolites [15,17,18,19]. For example, under certain synthesis conditions, it is possible to grow a layered material in which inorganic layers of ZSM-5 zeolite and organic layers consisting of ordered CTAB molecules alternate. Under the conditions generally used for zeolite synthesis (100–180 °C and high pH), the CTAB molecules do not decompose, but interact strongly with the components of aluminosilicate gel. As a result of using CTAB, it was possible to direct the synthesis towards the formation of inorganic-organic microporous materials and the design of hierarchical zeolite catalysts from a plate-like zeolite precursor, which opens up new possibilities for the complex production of mesoporous zeolites. The main factor in this process is the guest-host interactions between organic surfactant and inorganic framework during the self-assembly and structure evolution development [17,19]. In such a process, a swelling-type multilamellar ECNU-7P with alternative stacking of MWW nanosheets and organic CTAB layers was successfully prepared through a dissolution−recrystallization route. This was the first time that a simple surfactant CTAB and a layered zeolite precursor could act synergistically during self-assembly. As a result, an alternative, attractive pathway opens up to current post-synthetic approaches, or to the hydrothermal syntheses of MWW nanosheets with designed surfactants. Calcined Al-ECNU-7 turned into a hierarchical zeolite catalyst, and exhibited excellent activity, selectivity and stability during the catalytic conversion of bulky molecules. The present approach would be a general methodology and would be suitable for the direct synthesis of hierarchical layered zeolites with other topologies by controlling the self-assembly of a simple surfactant and zeolite precursor. More significantly, the low cost and commercial availability of the CTAB simple surfactant makes it more promising than the complex bifunctional surfactants currently used for the preparation of industrial heterogeneous catalysts.

Despite quite numerous studies of the morphology and catalytic properties of layered zeolites obtained by self-assembling method, studies of their local structure are not so widespread, although this is a key point for understanding of the catalytic activity of materials. Nuclear magnetic resonance (NMR) is one of the most versatile experimental methods to probe the local structure [20]; besides this technique enables to obtain at the microscopic level information on dynamics of intercalated species [21,22] and is successfully applied to study organic-inorganic layered materials [22,23,24,25].

Earlier, we reported on the results of the successful synthesis of 2D ZSM-5 and mordenite [26]. The aim of this work is by applying multinuclear NMR to follow up changes in the local structure at all the stages of preparation, starting from a freshly synthesized hybrid material, in which the confinement of organic and inorganic layers is implemented, then pillaring between the layers, and finally removal of organic material during calcination.

## 2. Results and Discussion

In this work, layered two-dimensional (2D) zeolites with mordenite and ZSM-5 structures were prepared and studied. Further in the text and in the figures, they will be denoted by three-letter structural codes adopted by the International Zeolite Association (IZA) [27], as MOR and MFI, using additional abbreviations those are marking a certain stage of preparation. Both materials were synthesized according to the procedure described in our previous work [26]. Pillaring of the obtained materials was done in accordance with the process proposed by Na et al. [28]. As a result, our method for preparing samples included four steps: (i) obtaining of organic-inorganic hybrid lamellar zeolites by self-assembling method with addition of CTAB (and tetrapropylammonium bromide (TPABr) as OSDA for the synthesis of 2D ZSM-5): the MOR-AS and MFI-AS samples; (ii) introduction of tetraethoxysilane (TEOS) molecules into the organic layer of the interlamellar space filled with CTAB molecules: the MOR-T and MFI-T samples; (iii) hydrolysis of an organosilicon compound and the formation of pillars of amorphous SiO_2_: the MOR-TH and MFI-TH samples; (iv) calcination to remove organic molecules: the MOR-P and MFI-P samples. A more detailed description of the preparation method can be found in Section 3.

### 2.1. X-ray Analysis

**Figure 1:**
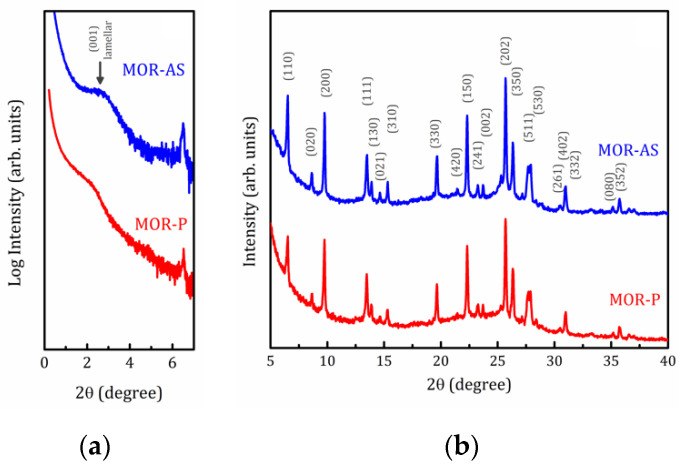
Small-angle (**a**) and full (**b**) XRD powder patterns for the mordenite samples as synthetized (MOR-AS) and pillared (MOR-P).

**Figure 2:**
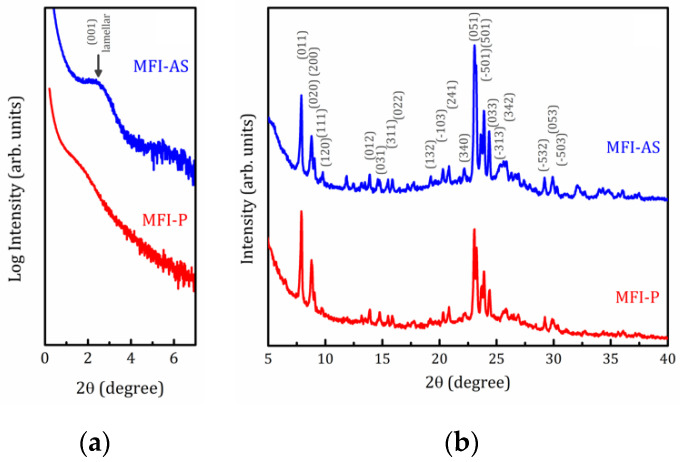
Small-angle (**a**) and full (**b**) XRD powder patterns for the ZSM-5 samples as synthetized (MFI-AS) and pillared (MFI-P).

Figure 1 and Figure 2 represent the X-ray Diffraction (XRD) patterns of both as synthetized and pillared MOR and MFI samples, respectively. As can be seen in Figure 1b and Figure 2b, both samples exhibit typical features of the corresponding zeolite structure with an amorphous halo (range 2θ between 17–30 degrees) which is very consistent with this kind of materials [28,29]. This amorphous halo should vanish after calcination process, but it is necessary to remind the amorphous character of the pillars even after calcination could maintain or intensify this feature.

Small angle X-ray scattering (SAXS) patterns shown in Figure 1a and Figure 2a unambiguously indicate the formation of lamellar mesophases [28]. For lamellar samples, the peak at 2θ = 2.7° for MOR-AS and 2θ = 2.3° for MFI-AS, corresponds to the (001) reflections with interplanar distances *d* = 3.2 and 3.8 nm, respectively. For pillared samples, this peak is smoothed and shifted toward small angles (2θ = 2.2 and 1.7° with *d* = 4.0 and 5.2 nm for MOR-P and MFI-P, respectively), which shows that the introduction of SiO_2_ pillars increased the interlamellar space. The present results are consistent with our previously reported data [26]. A wider interplanar distance *d* distribution is a clear evidence of the random growing of pillars, that is to say, some distances can be expanded while others can be contracted. A detailed discussion of the physical structure of these zeolite plates, separated by plates consisting of organic material, is of particular interest. However, this topic is beyond the scope of the present article and will be published separately elsewhere.

### 2.2. SEM-EDX Studies

**Figure 3:**
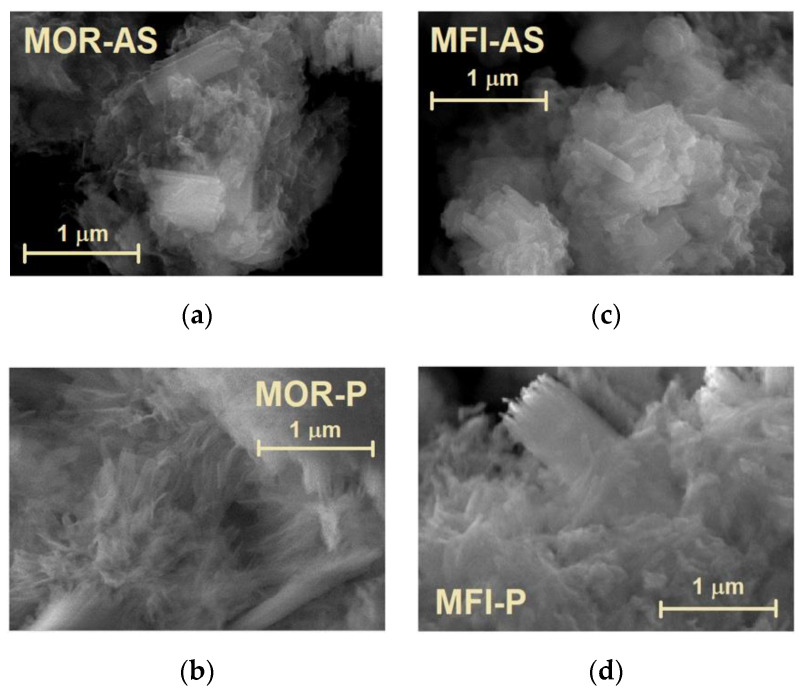
SEM images of the samples, as synthetized and pillared: MOR-AS (**a**), MOR-P (**b**), MFI-AS (**c**), MFI-P (**d**).

Figure 3 shows scanning electron microscopy (SEM) images for the initial as synthetized zeolites and the final samples after pillaring. As seen from Figure 3a,c both MOR-AS and MFI-AS have similar morphology: elongated plates up to 1 μm in length and 0.1 μm in width, combined in stacks. The pillaring does not change noticeably the morphology of the layered zeolites, see Figure 3b,d.

The results of the energy dispersive X-rays (EDX) elemental analysis of the as-synthetized and pillared materials are summarized in Table 1. Both MOR-AS and MFI-AS have nearly the same Si/Al ratios, 8.4 ± 0.3 and 8.8 ± 0.3, respectively. The Na/Al ratio in MFI-AS is close to unity within the experimental error, which means that all the negative charge due to partial substitution of Si for Al is compensated by Na^+^. In MOR-AS an excess of positive charge (Na/Al > 1) must be balanced by Br anions. No trace of Br^−^ was detected in the MFI set of samples and in MOR-P. This means that both CTAB and TPABr are present only in their cationic forms, CTA^+^ (hexadecyltrimethylammonium) and TPA^+^ (tetrapropylammonium), respectively, balancing the dangling bonds of the zeolite layers. Such a rather nontrivial question of the coordination of charged Al tetrahedra and organic cations requires additional research, which the authors plan to carry out in the future, and the results of which will be published elsewhere.

**Figure 4:**
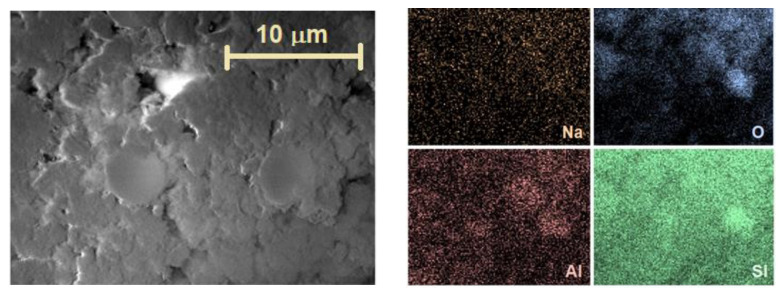
Element distribution maps in the MOR-P sample.

The pillaring results in an almost twofold increase in the Si/Al ratio, 15.2 ± 1.3 and 16.5 ± 1.0 for MOR-P and MFI-P, respectively, with a simultaneous decrease in sodium content, more pronounced for MOR-P. Sodium leaching is quite likely during sample processing in TEOS hydrolysis (Step 3). The role of compensating cations should eventually pass to protons, even if organic cations are involved in the hydrolyzed samples. In addition, it should be noted that all the materials are characterized by a certain inhomogeneity in the distribution of elements: there are regions with higher and lower Si/Al ratios, which is reflected in a rather large experimental error. An example of the element map distribution for the MOR-P sample is shown in Figure 4.

### 2.3. Thermal Analysis

**Figure 5:**
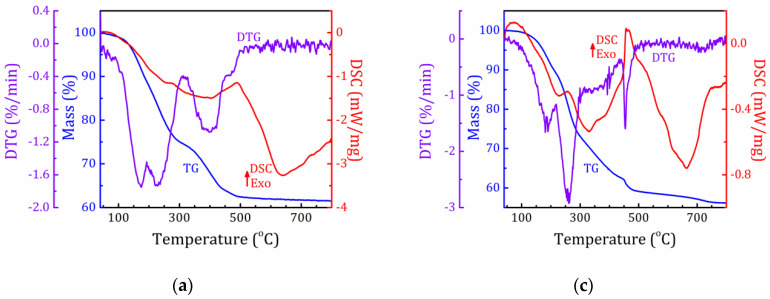

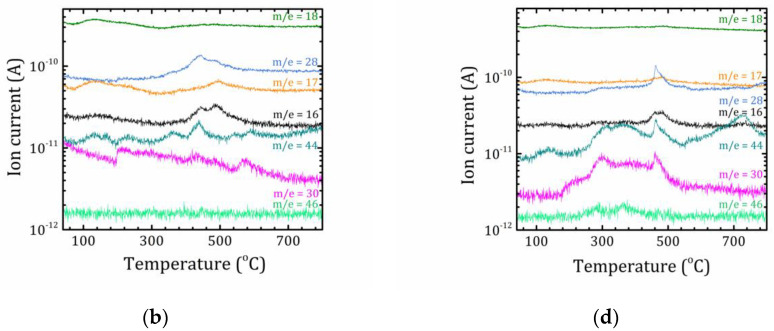
(**a**,**c**): TG (blue), DTG (violet), DSC (red) and (**b**,**d**): ion current (various colors) curves for the MOR-AS (**a**,**b**) and MFI-AS (**c**,**d**) samples.

The results of the simultaneous thermal analysis (including thermogravimetry (TG) and differential scanning calorimetry (DSC) combined with mass spectrometric analysis (MS) of the evolved gases) of the MOR-AS and MFI-AS samples are shown in Figure 5a–d, respectively.

Both samples exhibit a rather complex mass loss. For MOR-AS, the mass loss below 300 °C is associated with water release (*m*/*e* = 16, 17, 18) from macro- and microcavities, peaks at 175 and 228 °C, respectively. The mass loss between 300 and 500 °C is related with a multistep decomposition of CTA^+^ and PEG with formation of ammonium fragments, volatile low-carbon residues, e.g., ethylene (*m*/*e* = 28) and products of combustion of non-volatile high-carbon residues, CO_2_ and H_2_O. The peaks in the corresponding ion-current curves are accompanied by DSC peaks at 445 and 488 °C. In MFI-AS, water release occurs in one step below 220 °C, mass loss above 250 °C is associated with the decomposition of organic cations and molecules (CTA^+^, TPA^+^ and PEG).

### 2.4. NMR Study

#### 2.4.1. ^1^H MAS NMR and ^13^C CP-MAS NMR

**Figure 6:**
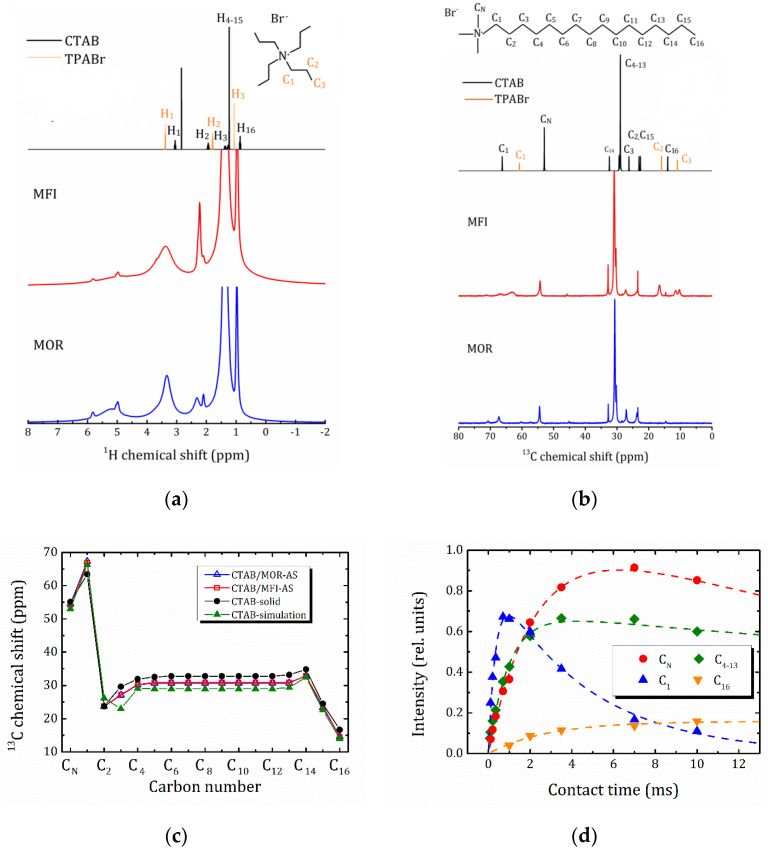
^1^H MAS NMR (**a**) and ^13^C CP/MAS NMR at *τ*_cp_ = 2 ms (**b**) spectra of the studied samples MOR-AS and MFI-AS with simulated spectra of CTAB and TPABr (at the top) given for a comparison; (**c**) ^13^C isotropic chemical shift profiles of CTAB: CTAB/MOR-AS (open triangles), CTAB/MFI-AS (open squares), crystalline powder from Ref. [23] (closed circles), simulated using www.nmrdb.org [30] (closed triangles); (**d**) integral peak intensity for C_N_, C_1_, C_4–13_ and C_16_ atoms of CTAB in MOR-AS versus contact pulse duration *τ*_cp_ (VCT experiment), dashed lines corresponds to the fitting within Equation (1).

Figure 6 shows the ^1^H MAS and ^13^C CP/MAS (at the contact pulse duration *τ*_cp_ = 2 ms) NMR spectra for the studied samples of “as-synthesized” MOR-AS and MFI-AS. All the spectral lines can be attributed to the organic molecules CTAB and TPABr, the latter for the MFI-AS sample only. The reference spectra of pure CTAB and TPABr substances, simulated using the online service www.nmrdb.org [30], as well as the spatial structure of their molecules with atom labeling, are shown in the upper part of Figure 6a,b. As can be seen, the spectra for both zeolite samples exhibit typical features of the corresponding organic molecules (except the ^1^H lines above 4.5 ppm that can be attributed to PEG and water), but all lines are broadened and shifted towards a lower magnetic field. The broadness of the spectral lines points out that the molecule mobility is frozen.

For better visualization, in Figure 6c the ^13^C chemical shifts for all carbon atoms of CTAB in the MOR-AS and MFI-AS samples are plotted versus the numbering of carbon atoms. The data for a crystalline CTAB powder from Ref. [23], together with the simulation for a CTAB molecule are given as a comparison. The ^13^C CTAB spectra for both MOR-AS and MFI-AS samples are very similar and correspond to immobilized rigid molecules in the all-trans conformation [23]. A chemical shift value of about 31 ppm can be attributed to C_4_–C_13_ carbon atoms in the central part of the CTAB chain. A typical value of the chemical shift obtained in crystalline *n*-alkanes in the *trans*-conformation is 33 ppm [31]; the lower chemical shift of these methylene carbons by 2 ppm is usually attributed to the presence of a significant fraction of gauche conformers [23]. A higher chemical shift of carbon C_1_ by 4 ppm evidences a strong interaction between the charged –[N(CH_3_)_3_]^+^ head of the CTAB and the zeolite framework. This is also confirmed by a linewidth of the ^1^H-NMR lines. The ^1^H NMR lines broaden as one moves from the tail towards the –[N(CH_3_)_3_]^+^ head of the molecule that means a decrease of the mobility of methyl groups (broad H_N_ peak) as compared to the CTAB tail. This is very consistent with the findings of studies on the inclusion of CTAB into a MWW type structure synthesized under basic/alkaline conditions, where it is suggested that CTAB can be included into hemicavities of MWW through intermolecular hydrogen bonding with bridged oxygen atoms that are connected to Q^4^ sites [19]. In the same way, in some NMR studies on the inclusion of Al into MCM-41 mesoporous aluminosilicates, which are synthesized using CTAB as a mesoporogen or structure directing agent, it was found that the polar head of CTAB shows a strong correlation with four-coordinated Al through electrostatic interactions between cationic ammonium-methyl head groups and tetrahedral Al (in the framework) [32]. In this sense, the interaction of the polar head of the surfactant with the surface of silica is carried out by silanol groups [33], which are very weak sites; while the interaction of CTAB with ordered aluminosilicate occurs by electrostatic interaction with the framework charge in the place where tetrahedral aluminum is present.

This conclusion is in agreement with the VCT experiment. Figure 6d represents the intensity of the selected carbon peaks of CTAB in MOR-AS as a function of contact time ^1^H-^13^C. For C_4–13_, the signal intensity is normalized per carbon nucleus.

The building up and loss of signal intensity during VCT can be described by the following Equation (1) [34]:

(1) $$I=I_{0}{\left(1-\frac{T_{\mathrm{CH}}}{T_{1\rho }}\right)}^{-1}\times \left[\exp\left(-\frac{\tau_{\mathrm{cp}}}{T_{1\rho }}\right)-\exp\left(-\frac{\tau_{\mathrm{cp}}}{T_{\mathrm{CH}}}\right)\right]$$

*T*_CH_ determines the rising part of the intensity and represents the efficiency of the cross polarization between ^1^H and ^13^C nuclei and is often related to the mobility of the nuclei under study: mobile atoms have a high *T*_CH_ value because of the inefficiency of cross-polarization. The decay of the signal is governed by the rate of ^1^H spin *T*_1ρ_ relaxation. The *T*_CH_ and *T*_1ρ_ parameters for the selected carbon sites of CTAB in MOR-AS, as determined from the dependencies shown in Figure 6d using Equation (1), are listed in Table 2. The C_N_ and the terminal methyl group (C_16_) have a much longer *T*_CH_ than other carbon atoms. The terminal C_16_ group has the greatest mobility: the largest *T*_CH_, and within the studied contact time range, the signal intensity does not decrease even at the longest *τ*_cp_ values that point out to a large *T*_1ρ_ value that cannot be determined due to the low signal intensity. The C_1_ group has the shortest *T*_CH_ and *T*_1ρ_ times and hence the lowest mobility.

**Figure 7:**
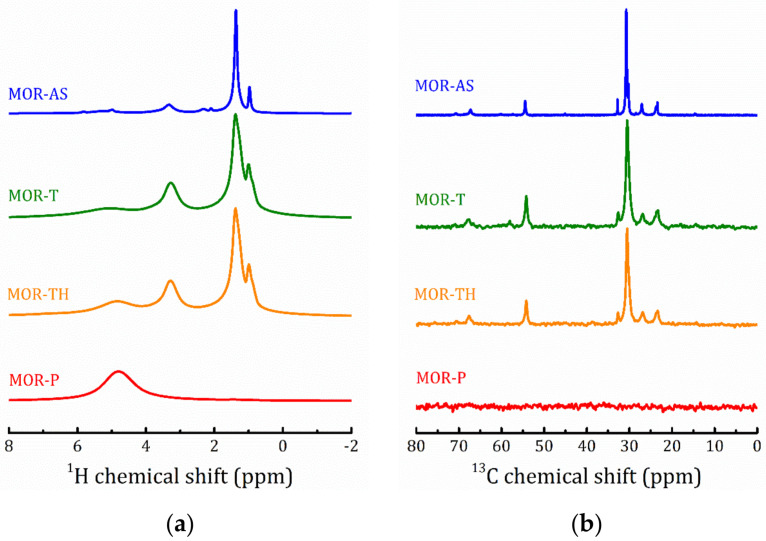

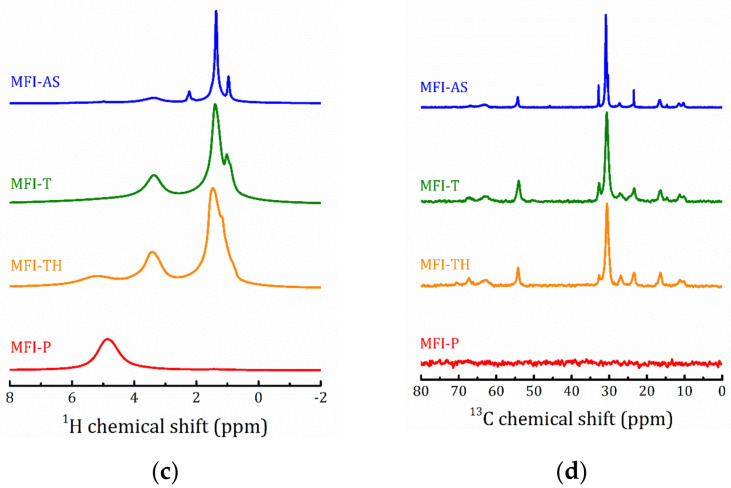
^1^H MAS NMR (**a**,**c**) and ^13^C CP/MAS NMR at *τ*_cp_ = 2 ms (**b**,**d**) spectra of the studied MOR (**a**,**b**) and MFI (**c**,**d**) samples at all the preparation steps.

Coming back to the NMR spectral analysis, the introduction of TEOS and subsequent hydrolysis result in an essential broadening of the ^1^H- and ^13^C-NMR lines that is associated with a further decrease in the mobility of CTAB, see Figure 7.

The line positions in MOR-T, MOR-TH, MFI-T and MFI-TH remain untouched as compared to MOR-AS and MFI-AS, respectively. Annealing results in the complete disappearance of organic matters (there are no traces of the ^13^C-NMR signal in the MOR-P and MFI-P spectra). A broad ^1^H line at 4.8 ppm points out to the presence of water molecules with restricted mobility.

#### 2.4.2. ^27^Al and ^29^Si MAS NMR

**Figure 8:**
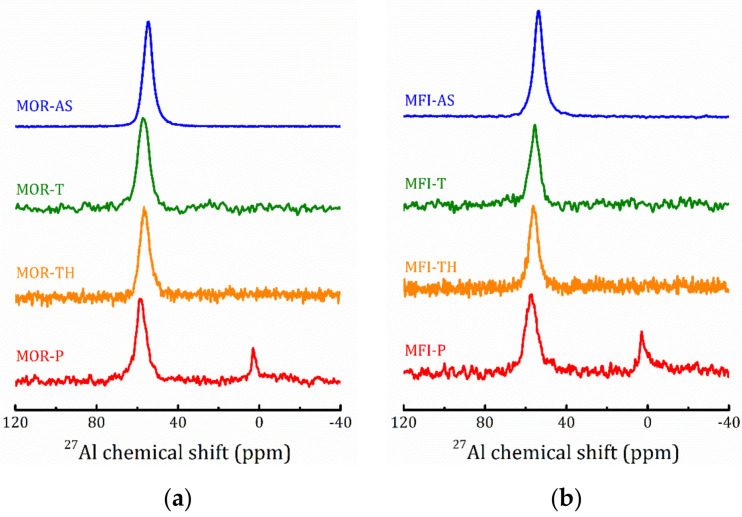
^27^Al MAS NMR spectra of the studied MOR (**a**) and MFI (**b**) samples at all the preparation steps.

The ^27^Al MAS NMR spectra confirm the regularity of the zeolite frameworks of the as prepared samples, see Figure 8, the only line at about 54 ppm corresponds to Al in regular tetrahedral sites. The introduction of TEOS and the subsequent hydrolysis procedure do not perturb much the framework aluminum: the line slightly broadens and shifts at 2–3 ppm due to interaction with TEOS, see Table 3. However, the calcination procedure results in the appearance of six-coordinated extra-framework Al (the line at about 2 ppm) (20 and 27% for MOR-P and MFI-P, respectively). The presence of six-coordinated Al is often observed in protonated zeolites obtained by calcination of the ammonium form [35,36,37,38].

It should be noted that the data reported in Table 3 can be used for a rough estimate of the ^27^Al MAS NMR spectra that was done by a simple fitting by a Lorentzian line. But, as one can see from Figure 8, after hydrolysis, a shoulder at about 52 ppm appears (more pronounced for pillared samples). Such an asymmetric shape of the spectral line is issued by quadrupole interactions [39] and may point out at an increase in the quadrupole interactions due to the deformation of [AlO_4_]^−^ tetrahedra [40,41].

**Figure 9:**
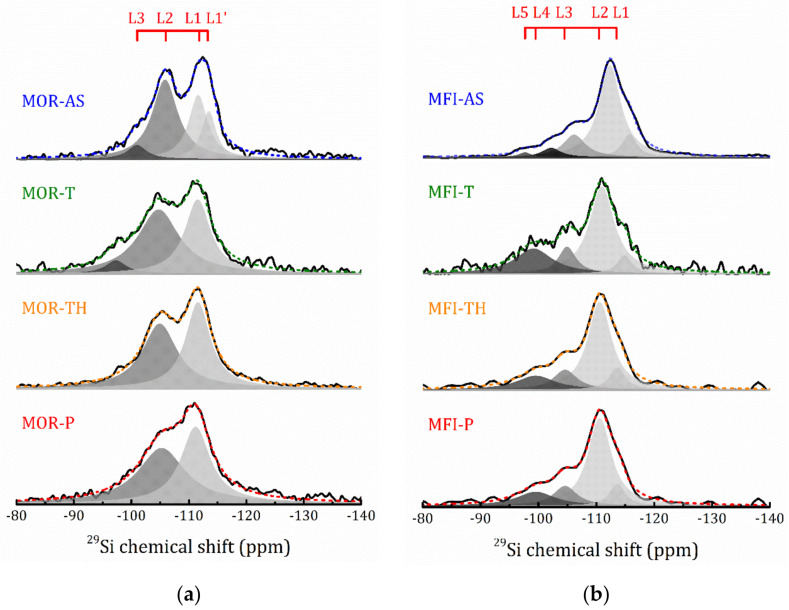
^29^Si MAS NMR spectra for the MOR (**a**) and MFI (**b**) samples at the all preparation steps. Filled patterns represent decomposition on Lorentzian functions, dashed lines represent the total fit.

Figure 9 represents the ^29^Si MAS NMR spectra of all the studied compounds. The spectra for the as-synthesized layered zeolites exhibit features typical for 3D zeolites.

For MOR-AS, the ^29^Si spectrum was fitted by four Lorentzian lines (L1, L1′, L2 and L3) that can be assigned with specific Q-type Si sites in the mordenite lattice: −113.5 and −111.6 ppm correspond to two different Q^4^(0Al) sites, whereas −105.8 and −101.0 ppm can be attributed to Q^4^(1Al) and Q^4^(2Al), respectively [42]. Using the integrated areas of these lines, the Si/Al ratio can be estimated as 8.3 [43,44]. This is in a fair agreement with the EDX data and implicitly confirms the ^29^Si NMR line assignment. However, rather important contributions of Q^4^(1Al) and Q^4^(2Al) are observed in layered mordenite, as compared with 3D mordenites with a close Si/Al ratio [45,46]. This could be due to the interaction of CTAB heads with Al tetrahedra at the interface that is truncated, resulting in a higher prevalence of Al in certain preferred sites of the zeolitic structure. In addition, one should take into account the change in the T-O-T angles for the Q^4^(*n*Al) sites due to local structural distortions in the 2D plate. For several zeolites, including mordenite, there is an almost linear correlation between the ^29^Si chemical shift of the Q^4^(*n*Al) signal with the magnitude of T-O-T angle [47]. And finally, there may be an effect of the simultaneous action of these two factors.

**Figure 10:**
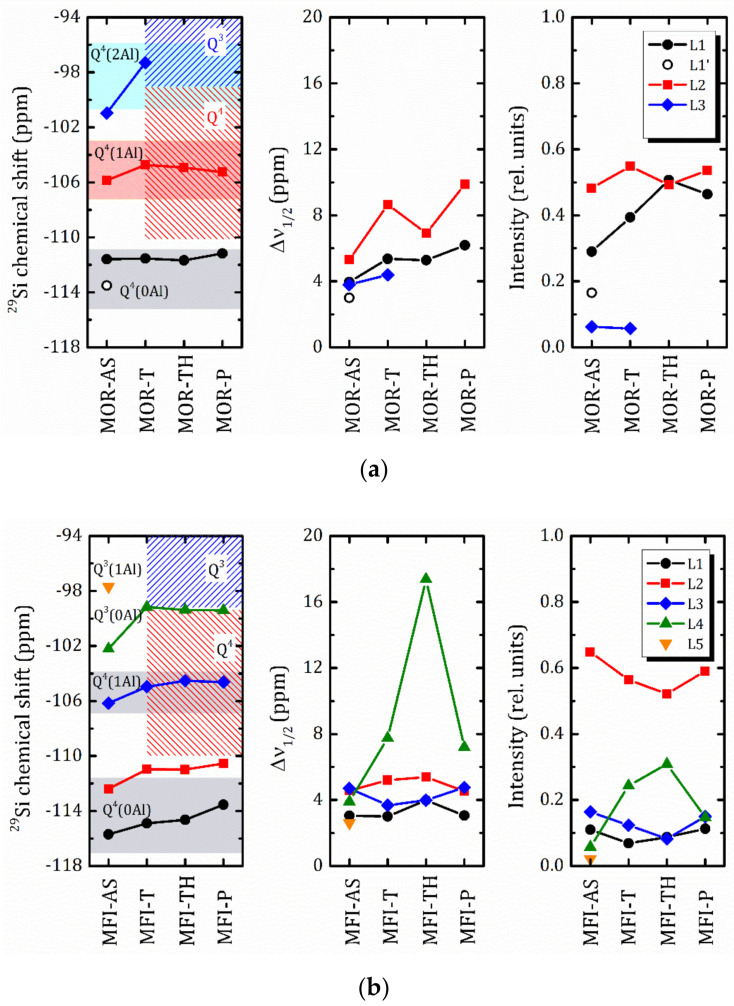
^29^Si NMR chemical shift (left), the line width at half maximum, Δν_1/2_ (center) and relative integral intensities (right) of the individual Lorentzian lines for the MOR (**a**) and MFI (**b**) samples at the all preparation steps. Filled areas show the typical ranges of ^29^Si chemical shift in zeolites (solid fill) and TEOS (hatched fill).

To follow the changes in the ^29^Si spectra that occur at each preparation step, in Figure 10 we plotted the parameters of individual spectral lines shown in Figure 9. As can be seen, for the mordenite sample, the introduction of TEOS (MOR-T) results in essential broadening the line width, the two lines previously attributed to Q^4^(0Al) are merged, and a low chemical shift part of the ^29^Si spectrum becomes more pronounced. After hydrolysis (MOR-TH) the spectrum can be perfectly fitted by two Lorentzian lines. Further, annealing (MOR-P) results in a slight line broadening and redistribution of the line intensities. The main difficulty in assigning the spectral line is caused by the partial overlapping of the Q^4^(1Al) and Q^4^(2Al) ^29^Si mordenite signal with Q^4^ and Q^3^ of TEOS. The typical ranges of ^29^Si chemical shift in mordenite [42,44], and TEOS [48] are shown in Figure 10a.

It should be noted that the ^29^Si signal below 90 ppm was not detected even in CP/MAS mode (see Figure S1 in Supplementary Materials). In MOR-T, after the introduction of TEOS, this means that from the first moments a sol-gel reaction (hydrolysis and polycondensation) takes place, which triggers the formation of a three-dimensional network of Si tetrahedra in the interlayer space of the zeolite, between the CTAB molecules. This is consistent with the data for gelled TEOS reported in [48]. The next step, a targeted hydrolysis procedure, completes it (total disappearance of the Q^3^ signal). A consistent increase in the intensity of the L1 line at each step of preparation implicitly points out the formation of SiO_2_ oligomers. And in the pillared MOR-P signal at −111.2 ppm corresponds to the overlapping mordenite Q^4^(0Al) site and Q^4^ of amorphous SiO_2_ [49,50].

In the layered ZSM-5 zeolite, MFI-AS, the ^29^Si spectrum can be decomposed into five Lorentzian lines (L1–L5), Figure 9b. The signals at −115.7 and −112.4 ppm can be attributed to two different Q^4^(0Al) sites, the remaining signals, −106.16, −102.2 and −97.7 ppm, can be assigned with Q^4^(1Al), Q^3^(0Al) and Q^3^(1Al), respectively [44,47,51]. The introduction of TEOS, in MFI-T, leads to significant changes in the Q^3^(0Al) signal: an increasing of the linewidth and integral intensity is observed, and the chemical shift is 3 ppm lower, Figure 10b. Overlapping basically triggers these changes with the Q^3^ signal of TEOS. Similarly to MOR-T, an autohydrolysis and condensation also occur in MFI-T, but with the predominant formation of Q^3^ structures. The subsequent hydrolysis procedure (MFI-P) leads to a further increase in the formation of Q^3^, but after annealing (MFI-P), a sharp decrease in the L4 line intensity means that this is due to Q^3^(0Al) of the ZSM-5 zeolite. The autohydrolysis is in good agreement with the ease of hydrolysis of this type of alkoxide compounds when exposed to a small amount of water or humidity. Step 1 was carried out in the presence of water in the reaction mixture and flushing with methanol of this zeolite/organics hybrid interlayered compound under reflux did not completely remove the water. In addition, the interlaminar diffusion treatments with TEOS (Step 2) were not carried out in a controlled dry atmosphere, so the presence of internal water in the sample and ambient humidity may cause autohydrolysis of the compound even before it is treated in water at 90 °C (Step 3).

## 3. Materials and Methods

Both sets of materials were synthesized according to the procedure described in our previous work [26]. Pillaring of the obtained materials was done in accordance with the process proposed by Na et al. [28].

Step 1: For mordenite, the organic components (3.123 g of CTAB, 0.5205 g of polyethylene glycol (PEG) 20000) and 0.47 g of NaOH were completely dissolved in 36.3 mL of H_2_O. After that 21.46 g of sodium silicate solution (25 wt% of SiO_2_ and 10.6 wt% of Na_2_O) were added. The obtained mixture was vigorously stirred for 20 min. Then, the solution of sodium aluminate (0.48 g of NaAlO_2_ dissolved in 26.6 g of H_2_O) was added dropwise. Finally, 26 g of a 10 wt% H_2_SO_4_ solution was added under vigorous stirring. The same method was used to obtain ZSM-5 zeolite, but with the addition of 2.66 g of TPABr as OSDA to other organic components.

The obtained mixtures were heated at 150 °C for 4 days in a stainless-steel autoclave with teflon coating under autogenous pressure. Then, the samples were filtered and washed with distilled water, and then washed with methanol under refluxing for 12 h at 60 °C to remove physically occluded surfactants. The resulting samples were labeled as MOR-AS and MFI-AS.

Step 2: 1.0 g of MOR-AS (or MFI-AS) sample was stirred in 5.0 g of TEOS for 12 h at 25 °C. Then samples were filtered and dried at 35 °C for 12 h. The obtained samples were labeled as MOR-T and MFI-T.

Step 3: To hydrolyze TEOS, 1.0 g of the MOR-T and MFI-T samples were stirred in 10.0 g of distilled water at 90 °C for 12 h. Washed with distilled water, filtered, and dried at 120 °C samples were labeled as MOR-TH and MFI-TH.

Step 4: Samples of MOR-TH and MFI-TH obtained after hydrolysis were calcined at 550 °C for 4 h in air to remove organic compounds. As a result, samples of pillared MOR and MFI were obtained, which were labeled MOR-P and MFI-P, respectively.

Powder XRD analysis was done on a Bruker D8 DISCOVER diffractometer using monochromatic CuK_α_ radiation (λ = 0.154056 nm). Diffractograms were recorded in the 2θ range of 5–40° (step width 0.0302°), where the main characteristic peaks of the MOR and MFI zeolites appear. SAXS patterns were recorded in a scan range from 0.2 to 7.0 2θ degree, step width 0.01°.

Simultaneous thermal analysis was carried out using a Netzsch STA 449 F1 Jupiter coupled with a QMS 403 Aëolos quadrupole mass spectrometer. The mass change of the samples and the composition of the evolved gases were registered. Analysis of samples was carried out in the temperature range 40–820 °C at a heating rate of 10 °C/min in an argon stream at a rate of 90 mL/min.

The morphology and elemental analysis of the samples was studied by an optical system integrated into D8 DISCOVER spectrometer (Bruker AXS, Karlsruhe, Germany) and by SEM applying Zeiss Merlin (Zeiss, Oberkochen, Germany) equipped with an EDX Oxford Instruments INCAx-act.

NMR spectra were recorded using a Bruker Avance IIIWB 400 MHz (Bruker, Karlsruhe, Germany) solid-state NMR spectrometer (operating with Topspin version 3.2) using a double-resonance 4 mm Magic Angle Spinning (MAS) probe. The operating frequencies were 400.23, 100.64, 104.28 and 79.5 MHz for ^1^H, ^13^C, ^27^Al, and ^29^Si nuclei, respectively. The rotor speed was 14 kHz. For all nuclei except ^13^C, the direct excitation method was used. To increase the intensity of ^13^C-NMR spectra, the cross-polarization (CP/MAS) method was applied. Variable contact time (VCT) experiments were performed with contact time *τ*_cp_ varied between 70 and 10,000 µs. The relaxation delay time was 5 s. Tetramethylsilane (TMS) was used as an external standard.

## 4. Conclusions

Mesostructured zeolite materials with the crystalline structure of MOR and MFI having Si/Al ratios equal to 8.4 and 8.8, respectively, were grown in the form of lamellar inorganic phases separated by layers of organic material. CTAB was used as an agent that creates mesopores, in a one-pot synthesis. It was shown that the mesostructured array consists of alternating lamellas of CTAB, with a thickness of ~3.5 nm, and a zeolite, with a thickness of one-unit cell along the z axis for each of the synthesized structures. Both lamellar zeolites have a similar morphology: elongated plates up to 1 μm long and 0.1 μm wide combined in stacks.

^27^Al and ^29^Si MAS NMR spectra confirm the regularity of the zeolite frameworks of the as-synthetized layered 2D materials: there is no extra-framework Al, ^29^Si spectra correspond to bulk 3D MOR and MFI with broadened lines from Q^4^(0Al), Q^4^(1Al) and Q^4^(2Al) sites.

Analysis of the ^1^H and ^13^C MAS NMR spectra and the VCT experiment evidence a strong interaction between the charged –[N(CH_3_)_3_]^+^ heads of CTAB and the zeolite framework: the C_1_ and terminal C_16_ groups of CTAB have the lowest and highest mobility, respectively. Since in the both MOR-AS and MFI-AS samples the Na/Al ratio is close to unity (a slight excess of Na^+^ found in MOR-AS is balanced by Br^−^ anions), CTA^+^ and TPA^+^ cations balance the dangling bonds of the zeolite layers.

The introduction of TEOS from the beginning leads to autohydrolysis and the formation of SiO_2_ oligomers due to the water contained in the sample. Further targeted hydrolysis completes the formation of amorphous SiO_2_ pillars separating the zeolite layers and holding them at fixed distances after thermal removal of the organic layers. Annealing leads to a partial drop out of Al from the zeolite frameworks (the appearance of extra-framework six-coordinated Al species). This implicitly points out that CTA^+^ cations in the as-synthetized materials are localized near [AlO_4_]^−^, and the removal of organic cations leads to a partial collapse of the local structure. However, in general, the zeolite structure of the layers is preserved. After calcination the role of compensating cations should eventually pass to protons; moreover, the surface hydroxyls should balance of the dangling bonds of the zeolite layers. From this perspective the study of the inner surface of the pillared zeolites is of great interest and is actually under evaluation.

## Figures and Tables

**Table 1:** EDX elemental analysis of the as synthetized and pillared samples.

Sample	Na/Al	Si/Al	Br/Al
MOR-AS	1.14 ± 0.03	8.4 ± 0.3	0.24 ± 0.05
MOR-P	0.17 ± 0.06	15.2 ± 1.3	Not detected
MFI-AS	1.11 ± 0.09	8.8 ± 0.4	Not detected
MFI-P	0.34 ± 0.05	14.8 ± 1.0	Not detected

**Table 2:** Values of *T*_CH_ and *T*_1_ρ for the selected CTAB spectral lines in MOR-AS derived from ^1^H-^13^C CP/MAS NMR measurements.

CTAB Carbon Site	δ (ppm)	*T*_CH_ (ms)	*T*_1ρ_ (ms)
C_N_	54.5	2.21 ± 0.04	31 ± 13
C_1_	67.3	0.37 ± 0.04	4.5 ± 0.5
C_4__–_13__	30.5	0.98 ± 0.12	70 ± 30
C_16_	14.6	2.8 ± 0.3	-

**Table 3:** ^27^Al NMR chemical shift (ν_0_) and line width at half maximum (Δν_1/2_) in the studied samples.

Sample	ν_0_ (ppm)	Δν_1/2_ (ppm)	Sample	ν_0_ (ppm)	Δν_1/2_ (ppm)
MOR-AS	54.6 ± 0.1	4.8 ± 0.1	MFI-AS	53.7 ± 0.1	4.9 ± 0.1
MOR-T	56.9 ± 0.1	5.6 ± 0.1	MFI-T	55.9 ± 0.1	4.9 ± 0.1
MOR-TH	56.3 ± 0.1	5.0 ± 0.1	MFI-TH	56.1 ± 0.1	4.7 ± 0.1
MOR-P	58.1 ± 0.1 2.6 ± 0.1	5.7 ± 0.1 4.2 ± 0.2	MFI-P	57.2 ± 0.1 1.6 ± 0.1	6.2 ± 0.1 8.5 ± 0.1

## Supplementary Materials

The following is available online, Figure S1: ^29^Si {^1^H} CP/MAS NMR at *τ*_cp_ = 2 ms for the MOR-T and MFI-T samples.
