# Supplementary material for: Local Structures of Two-Dimensional Zeolites—Mordenite and ZSM-5—Probed by Multinuclear NMR
Source: Molecules. 2020 Oct 14;25(20):4678. doi: 10.3390/molecules25204678 (PMC7587376; doi:10.3390/molecules25204678)
Supplement: Supplementary file 1 [file molecules-25-04678-s001.pdf]

Supplementary materials

Local structures of two-dimensional zeolites – mordenite and ZSM-5 probed by multinuclear NMR

Marina G. Shelyapina[1,*], Rosario I. Yocupicio-Gaxiola[2], Yulia V. Zhelesniak[1], Mikhail Chislov[1], Joel Antúnez-García[3], Fabian N. Murrieta-Rico[3], Donald Homero-Galván[3], Vitalii Petranovskii[3], Sergio Fuentes Moyado[3]

[1]Saint-Petersburg State University, 7/9 Universitetskaya nab., St. Petersburg 199034, Russia;
[2]Centro de Investigación Científica y de Educación Superior de Ensenada, Apdo. 22860, Ensenada, B.C., México
[3]Centro de Nanociencias y Nanotecnología, Universidad Nacional Autónoma de México, Ensenada 22860, Baja California, México

* Correspondence: Department of Nuclear Physics Research Methods, 1, Ulyanovstaya st., Peterhof, 199504, Saint Petersburg, Russia; e-mail: marina.shelyapina@spbu.ru

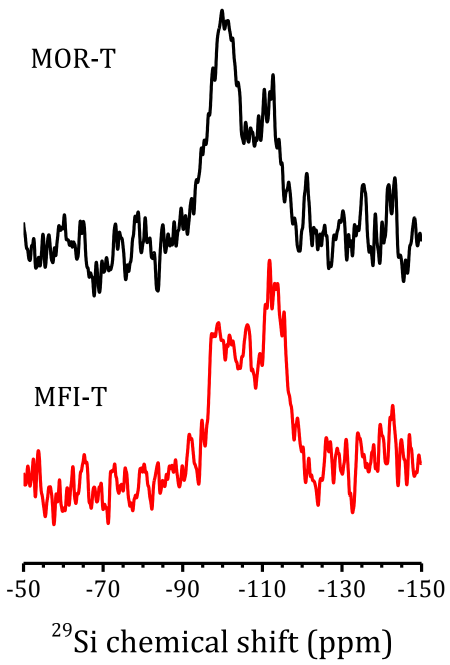

Figure S1. $^{29}Si\{^1H\}$ CP/MAS NMR at τ_{cp} = 2 ms for the MOR-T and MFI-T samples.
